# Therapeutic Development for CGG Repeat Expansion-Associated Neurodegeneration

**DOI:** 10.3389/fncel.2021.655568

**Published:** 2021-05-12

**Authors:** Keqin Xu, Yujing Li, Emily G. Allen, Peng Jin

**Affiliations:** ^1^Department of Human Genetics, School of Medicine, Emory University, Atlanta, GA, United States; ^2^Department of Neurology, Xiangya Hospital, Central South University, Changsha, China

**Keywords:** FXTAS, NIID, ET, RNA dysregulation, sequestration, RNA binding proteins, miRNA, therapeutic strategies

## Abstract

Non-coding repeat expansions, such as CGG, GGC, CUG, CCUG, and GGGGCC, have been shown to be involved in many human diseases, particularly neurological disorders. Of the diverse pathogenic mechanisms proposed in these neurodegenerative diseases, dysregulated RNA metabolism has emerged as an important contributor. Expanded repeat RNAs that form particular structures aggregate to form RNA foci, sequestering various RNA binding proteins and consequently altering RNA splicing, transport, and other downstream biological processes. One of these repeat expansion-associated diseases, fragile X-associated tremor/ataxia syndrome (FXTAS), is caused by a CGG repeat expansion in the 5’UTR region of the fragile X mental retardation 1 (*FMR1*) gene. Moreover, recent studies have revealed abnormal GGC repeat expansion within the 5’UTR region of the *NOTCH2NLC* gene in both essential tremor (ET) and neuronal intranuclear inclusion disease (NIID). These CGG repeat expansion-associated diseases share genetic, pathological, and clinical features. Identification of the similarities at the molecular level could lead to a better understanding of the disease mechanisms as well as developing novel therapeutic strategies. Here, we highlight our current understanding of the molecular pathogenesis of CGG repeat expansion-associated diseases and discuss potential therapeutic interventions for these neurological disorders.

## Introduction

**Figure 1:**
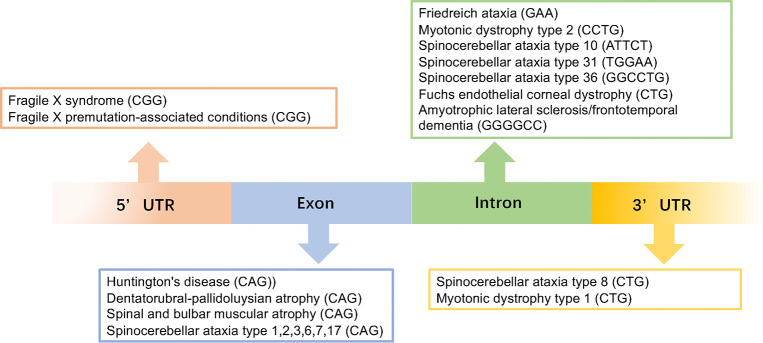
This image gives an overview of the repeat expansions seen in different diseases with the gene locations for each repeat.

Microsatellite repeat expansions, also known as simple sequence repeats (SSR) or short tandem repeats (STR), are short repeat sequences consisting of 3–6 nucleotides that comprise around 3% of the human genome (Ellegren, 2004; Mirkin, 2007; Richard et al., 2008). They can occur in coding regions, intronic regions, 5’ and 3’ untranslated regions (UTRs), and intergenic regions (Toth et al., 2000; Payseur et al., 2011; Figure 1). The repeat length is also highly variable and can range from hundreds to a few thousand (Zhang and Ashizawa, 2017). Thus far, over 40 neurodegenerative diseases have been reported to be caused by the expansion of an unstable repeat sequences in the associated genes.

Fragile X-associated tremor/ataxia syndrome (FXTAS) is one of these repeat expansion disorders characterized by a CGG or GGC repeat expansion (55–200 repeats) in the 5′ UTR of the Fragile X Mental Retardation 1 (*FMR1*) gene (Hagerman et al., 2001). Clinical features include progressive gait ataxia, intention tremor, cognitive decline, and Parkinsonism (Berry-Kravis et al., 2007). The carrier frequency of the CGG premutation is 1 in 813 males and 1 in 259 females in the general population, and it is estimated that 1 in 3,000 males from the general population will have a risk of developing FXTAS (Dombrowski et al., 2002; Jacquemont et al., 2004). In contrast to FXTAS, those carrying CGG repeats greater than 200 in the *FMR1* gene can present with fragile X syndrome (FXS), another neurodegenerative disease that has different clinical features. The clinical manifestations of FXS include delayed development of speech and language, increased susceptibility to seizures, anxiety, and hyperactive behavior (Hagerman et al., 2017). Unlike FXTAS, the larger number of CGG repeats in FXS leads to DNA methylation and transcriptional gene silencing, resulting in the loss of fragile X mental retardation protein (FMRP).

In addition to fragile X-associated disorders, recent studies have advanced our understanding of CGG or GGC repeat expansions in neurodegenerative disease. A flurry of recent exciting studies have revealed that the expanded GGC repeat, located in the 5′ UTR of the *NOTCH2NLC* gene, is a causative genetic contributor to *NOTCH2NLC*-related repeat expansion disorders, including neuronal intranuclear inclusion disease (NIID), Alzheimer’s disease, Parkinson’s disease, essential tremor (ET), leukoencephalopathy, and frontotemporal dementia (Deng et al., 2019; Ishiura et al., 2019; Okubo et al., 2019; Sone et al., 2019; Tian et al., 2019; Jiao et al., 2020; Ma et al., 2020; Sun et al., 2020). Neuronal intranuclear inclusion disease (NIID) is a neurodegenerative disease characterized by the widely distributed eosinophilic hyaline intranuclear inclusions in the central nervous system and peripheral nervous system. Clinically, NIID presents with a broad spectrum of manifestations, including dementia, autonomic dysfunction, cerebellar ataxia, parkinsonism, and peripheral neuropathy (Sone et al., 2016). Aside from the presence of CGG/GGC repeat expansions that have been found in both NIID and FXTAS, they share some striking similarities on clinical characteristics (Gelpi et al., 2017; Lim et al., 2020). First, tremor and ataxia are the common clinical features in patients with NIID and FXTAS. Second, brain MRI showed the same change of T2-weighted hyperintensity areas in the middle cerebellar peduncles (MCP sign; Ishiura et al., 2019). Furthermore both have the hallmark feature of eosinophilic intranuclear inclusions in the brain and non-neuronal tissues (Haltia et al., 1984; Hunsaker et al., 2011; Sone et al., 2011). These shared characteristics suggest that these CGG repeat expansion-associated diseases may share a common pathologic mechanism due to the neuronal toxicity caused by CGG repeats. Therefore, a better understanding of the molecular pathogenesis of these diseases will facilitate further development of novel therapeutic strategies.

Because NIID and its related disorders have only drawn increased attention within the last 2 years, there has not been considerable research done to understand the mechanism of disease, let alone identification of potential therapies. Based on the similarities shared by these CGG/GCC repeat expansion disorders, using the existing knowledge of CGG repeat expansions in FXTAS could provide meaningful references. Based on FXTAS research, two major mechanisms have been proposed: RNA gain-of-function and repeat-associated non-AUG (RAN) translation (Jin et al., 2007; Sofola et al., 2007; Sellier et al., 2013; Todd et al., 2013). The presence of both toxic mRNA and an FMRpolyG peptide have been confirmed in human and animal models, and both may have an effect on disease pathogenesis. So far, there is not a consensus, but the current therapies are based on these two mechanisms. In this review article, we will outline the latest advances in the mechanisms of CGG repeat expansion-associated diseases, particularly FXTAS and *NOTCH2NLC*-related disorders, with a main focus on the development of therapeutic strategies.

## Toxic Gain-of-Function Mechanism Caused By Expanded CGG Repeats

Repeat-containing RNAs with toxic gain-of-function generated from the repeat expansions in the non-coding regions of the gene(s) have been linked to diverse human diseases, including myotonic dystrophy type 1 (DM1), myotonic dystrophy type 2 (DM2), Spinocerebellar Ataxia Type 8 (SCA 8), and FXTAS (La Spada and Taylor, 2010; Todd and Paulson, 2010). The RNA toxicity mechanism depends on the repeat size, repeat location, the RNA-binding proteins that are sequestered by the repeat-containing RNAs, and their translated products. Theoretically, the expanded repeat RNA can form special structures and aggregates to form RNA foci. The repeat RNA within these foci will directly interact with repeat RNA-binding proteins (RBPs) and thereby sequestrating their normal function, which leads to downstream alterations, resulting in cellular toxicity.

Our current understanding of the CGG repeat RNA toxicity in FXTAS pathogenesis comes from multiple studies of human post-mortem tissues and experimental animal models. CGG-containing RNA levels are increased up to eight-fold while protein levels remain normal or slightly reduced in patients (Kenneson et al., 2001). To determine the gain-of-toxicity mechanism from the CGG repeats, a set of studies with *Drosophila* and mice showed deleterious neurodegeneration with the expression of expanded CGG repeats (Jin et al., 2003; Hukema et al., 2014). Further, studies of post-mortem FXTAS brains revealed the presence of CGG-containing transcripts in ubiquitinated nuclear inclusions. These findings strongly support the significance of CGG repeat RNA in a toxic gain-of-function mechanism.

The expanded CGG repeats can form a hairpin structure that creates excess binding sites for RBPs, sequestering them from interacting with other substrates, causing downstream biological effects and contributing to pathology. Various RBPs have been reported in human, fly and mouse FXTAS models, such as Pur-alpha (Pura), Heterogeneous nuclear ribonucleoproteins A2/B1 (hnRNPA2/B1), Muscleblind Like Splicing Regulator 1 (MBNL1), SRC associated in mitosis of 68 kDa (Sam68), CUG-binding proteins (CUGBP1), Heterogeneous nuclear ribonuclearproteins (hnRNP G), and Drosha and DiGeorge syndrome critical region 8 complex (Drosha-DGCR8; Jin et al., 2007; Sofola et al., 2007; Sellier et al., 2010, 2013; Muslimov et al., 2011). Moreover, overexpression of most of these RBPs can suppress RNA toxicity and rescue the phenotype. For example, CGG repeats can sequestrate DGCR8 and DROSHA, which play an important role in microRNA biogenesis. Sequestration of DGCR8 and DROSHA by expanded CGG repeats expressed in cells lines and in human FXTAS brains could lead to a reduction of microRNAs generation. Studies have shown reduced neuronal cell viability in neuronal cells expressing CGG repeats; however, the overexpression of DGCR8 was able to rescue neuronal cell death (Sellier et al., 2013). Extending this RNA toxicity mechanism to other CGG/GGC repeat disorders like NIID, which contains a GGC repeat expansion in *NOTCH2NLC* gene, we could speculate that the GGC-containing transcripts may also exist in the ubiquitinated nuclear inclusions of NIID. Further, the GGC and CGG repeat RNAs could share the same secondary structures, recruiting similar RBPs into nuclear inclusions, preventing their normal functions in RNA localization and transport in neurons, and consequently leading to severe phenotypes.

In summary, the expression of the expanded CGG repeat transcripts significantly correlates with neurodegenerative phenotypes, although their contribution to the disease pathogenesis remains to be defined. Thus, further studies on repeat-containing RNA toxicity are needed to identify similarities and specificities for the various types of CGG repeat diseases.

## Repeat-Associated Non-AUG (RAN) Translation

In addition to the RNA gain-of-function mechanism, a newly recognized mechanism of RAN translation has been proposed. In RAN translation, proteins are synthesized directly from the expanded repeats in the absence of the canonical AUG start codon (Zu et al., 2011). RAN translation has been detected in many repeat expansion disorders (e.g., Amyotrophic Lateral Sclerosis and Frontotemporal Dementia (ALS-FTD) with expanded GGGGCC repeats, Spinocerebellar Ataxia Type 8 (SCA8) with CAG repeats, and FXTAS with CGG repeats (Zu et al., 2011; Sellier et al., 2013), suggesting an essential role of the shared RAN translation mechanism in disorders caused by various repeat expansion.

Using FXTAS as an example, researchers realized that previous findings of RNA toxicity mechanism alone could not fully account for the intranuclear inclusions in patients. In the FXTAS human brain, the large ubiquitin-positive aggregates are not only made of RBPs but rather of various proteins that do not interact directly with CGG-repeat mRNA (Greco et al., 2006; Iwahashi et al., 2006), which resemble the intranuclear inclusions in protein-mediated neurodegenerative disorders (Williams and Paulson, 2008). To understand this protein-mediated toxicity, significant effort has been made in the last decade. Abundant studies suggest that the CGG-repeat RNAs can be RAN translated into pathogenic polyglycine-containing proteins (FMRpolyG) that contain an expanded polyglycine stretch near the N-terminus and is directly linked to FXTAS. FMRpolyG, detected in the intranuclear inclusions, is a protein prone to aggregation. Expression of the FMRployG can alter the ubiquitin-proteasome system (UPS) in both cell and fly models (Boivin et al., 2018). Indeed, FMRpolyG has been confirmed to exist in FXTAS patient brains as well as fly and mouse FXTAS models (Todd et al., 2013). Furthermore, FMRpolyG has been directly linked to the CGG-repeat-associated toxicity in FXTAS mouse and *Drosophila* models. More specifically, FXTAS mice with expression of the 5′UTR of *Fmr1* show neuronal inclusion formation, Purkinje cell loss, and locomotor deficits, while mice expressing CGG repeat RNA without FMRpolyG did not exhibit neurodegeneration as measured with behavioral tests. Notably, overexpression of lamina-associated polypeptide 2 beta (LAP2β), a nuclear membrane protein that co-precipitated with FMRpolyG, could rescue neuronal cell death caused by FMRpolyG (Sellier et al., 2017). These findings significantly support the contribution of RAN translation to the pathogenesis of FXTAS.

Given that NIID patients have a similar size of GGC repeats embedded in the 5′UTR, which also forms the hairpin RNA structure which could enhance translation initiation at the near-cognate codons located upstream of the repeats (Todd et al., 2013), it is logical to predict that RAN translation may occur in NIID as well. Furthermore, a defining signature of NIID is the intranuclear inclusions in patient tissues. Interestingly, the intranuclear inclusions in NIID patients partially resemble those of FXTAS, which are both ubiquitinated and immunoreactive for p62 (Mori et al., 2012), supporting our prediction that NIID and FXTAS could share overlapping mechanisms of neurodegeneration characterized by formation of the neural inclusions. Studies of the inclusion components have confirmed the presence of N-Ethylmaleimide Sensitive Factor (NSF), Syntaxin-binding protein 1 (Unc18–1), Heat shock protein 90 (HSP90), and Dynamin-1-like protein (DNM1L) in intranuclear inclusions from NIID human brain tissue (Pountney et al., 2008). Based on the known inclusion components in FXTAS, it will be interesting to determine whether the mutant protein caused by RAN translation is also present in the intranuclear inclusions of NIID.

Thus far, much of what we knew comes from study of FXTAS models, motivating us to infer that other CGG repeat disorders may also undergo RAN translation, which contributes to neurodegeneration. However, whether CGG RNA toxicity or RAN translation of CGG repeats might contribute to pathogenicity in CGG repeat disorders other than FXTAS is not yet known, as the pathology of these repeat disorders at the molecular level are far more complex than originally thought.

## Therapeutic Development

Currently, no therapies are sufficient to cure CGG expansion disorders, and current clinical treatments are only able to relieve symptoms of patients. The progress in research into disease mechanisms serves as a bottleneck to facilitate the development of preventive and therapeutic interventions. Given that mutant RNA containing expanded CGG repeats can be pathogenic by sequestering RBPs as well as being translated into toxic proteins, several potential therapeutic approaches could be developed *via* targeting at the DNA, RNA, and protein levels. Specifically, blocking generation of or enhancing degradation of the repeat-containing RNAs, inhibiting the RNA-protein interactions, and reducing the toxic protein aggregation could open a new avenue for development of therapeutic strategies for FXTAS, NIID, and other disorders caused by CGG/GGC repeats. Below, we highlight some recent advances.

### Antisense Oligonucleotide Therapy

Antisense Oligonucleotides (ASOs) are a modified single-stranded nucleic acid that can bind to mRNA targets. It functions by altering the secondary structure of mRNA, which results in blocking the binding of RBPs or triggering RNaseH-mediated degradation of target RNAs (Bennett et al., 2019). Oligonucleotide-targeting strategies have been employed successfully to improve neurodegeneration in diverse repeat disorders (Smith and Zain, 2019). One encouraging example is amyotrophic lateral sclerosis (ALS). Research showed that ASOs targeting upstream of the GGGGCC repeats led to reduction of RNA foci, increased survival from glutamate excitotoxicity, and improvement of abnormal gene expression in fibroblast and induced pluripotent stem cell (iPSC)-derived neurons (Sareen et al., 2013; Abati et al., 2020). Further studies conducted in mouse models confirmed these results: ASOs targeting the repeat-containing RNAs reduced the formation of RNA foci and toxic proteins, thus alleviating the behavioral and cognitive deficits (McCampbell et al., 2018). Similarly, a breakthrough has been made in the targeted knockdown of the CAG repeat containing RNA using a synthetic gapmer oligonucleotide in the myotonic dystrophy I mouse model (Nguyen and Yokota, 2020).

Significantly, clinical trials have been conducted in patients (Miller et al., 2013; Tabrizi et al., 2019) using ASOs targeting superoxide dismutase 1 (SOD1) in ALS and the mutant huntingtin gene in Huntington’s disease. In addition, ASO-mediated protein therapy also holds great promise. This can be achieved by reducing the protein aggregation or promoting the degradation of aberrant repeat expanded proteins. One example has been used for the ALS-associated RBP, TAR DNA-binding protein (TDP-43). ASOs were successfully used to target ataxin-2, an RBP with multiple roles in RNA metabolism. ASOs targeting ataxin-2 in TDP-43 transgenic mice could reduce aggregation of TDP-43 protein, thus extending their lifespan and improving their motor function (Becker et al., 2017). Another group also identified that ASOs targeting ataxin 2 could suppress neurodegeneration in ALS and frontotemporal dementia (FTD) mediated by a GGGGCC hexanucleotide repeat expansion in patient-derived neurons (Zhang et al., 2018). Importantly, these results raise the possibility that ASO-mediated reduction of RBPs might be helpful in CGG repeat disorders with TDP-43 proteinopathy. Consistent with this hypothesis, a study in the FXTAS *Drosophila* model identified TDP-43 as a suppressor of CGG repeat-induced toxicity. The authors found that TDP-43 suppresses CGG-mediated toxicity through interactions with hnRNP A2/B1, thereby preventing the alterations in RNA splicing triggered by hnRNP A2/B1 sequestration (He et al., 2014). Thus, using ASOs targeting toxic aggregation-prone RBPs to modulate aggregation and protein degradation could be of therapeutic significance for various CGG repeat diseases.

While ASOs are highly promising for the development of therapeutic strategies, some issues have to be considered. Given that the CGG repeat structure has been proposed to be critical for the complex formation with oligonucleotides (Tran et al., 2014), this highly structured repeat expansion in the 5′ UTR could affect ASO affinity, potentially leading to the inefficient accumulation of ASOs in the target tissues. Moreover, although ASOs can efficiently diffuse through the central nervous system and have high intake by neuronal cells, the blood-brain barrier (Rinaldi and Wood, 2018) may severely hinder the delivery of ASOs, raising a key issue on how to improve efficiency of delivery. Finally, further efforts are needed to ensure that ASOs operate with CGG repeats without altering translation of the downstream open reading frame.

### RNA Interference (RNAi)

RNAi has also emerged as a promising approach to treat expanded repeat disorders. With RNAi, double-stranded RNA molecules bind to target mRNAs and specifically degrade mRNA and suppress protein expression (Castanotto and Rossi, 2009). MicroRNA (miRNA), small interfering RNA (siRNA) and short hairpin RNA (shRNA) have become the most common RNA molecules for RNAi.

In ALS, studies have shown that siRNA targeting the GGGGCC repeat-containing transcripts significantly reduced mutant mRNA and RNA foci formation in fibroblasts, iPSC-derived motor neurons, and in a mouse model (Hu et al., 2017; Martier et al., 2019a, b). Similarly, in Huntington’s disease, which is caused by a CAG repeat expansion in the huntingtin gene (HTT), AMT-130, an AAV-delivered miRNA that targets Htt has been applied to reduce HTT levels successfully, leading to an improvement of neuropathology. In FXTAS, accumulating evidence from RNAi studies has shown that knockdown of *gypsy* (Tan et al., 2012) and TBPH (He et al., 2014) can suppress the neuronal toxicity caused by CGG repeats in a *Drosophila* model. However, little information is available for RNAi that directly targets the CGG repeat-containing RNAs.

Taken together, targeting CGG repeat RNAs by RNAi is expected to have therapeutic benefit in CGG repeat related disorders. However, there are still many technical issues to be studied. For instance, effective delivery of synthetic siRNAs or miRNAs into specific brain regions is challenging. RNAi systems are expected to be optimized not only to cross the blood-brain barrier but also efficiently target repeat expanded RNAs. However, the risk of off-target effects and immunogenicity should also be considered (Meng and Lu, 2017). As research progresses, advances in adeno-associated virus (AAV) vector usage have contributed substantially to the treatment of a variety of neurodegenerative diseases (Castanotto and Rossi, 2009). However, introduction of the virus into patients poses additional issues.

### Small Molecules

Emerging evidence has confirmed that some small molecules can regulate CGG expansion toxicity by interacting specifically with CGG repeat RNAs (Disney et al., 2012; Yang et al., 2016). They have a high affinity for CGG hairpins and can reduce the RBPs sequestration (Tran et al., 2014) by binding to the CGG repeat transcripts. Of the small molecules identified, 9-hydroxy-5,11-dimethyl-2-(2-(piperidin-1-yl)ethyl)-6H-pyrido[4, 3-b]carbazol-2-ium, is able to bind CGG repeats *in vitro*, improve FXTAS-associated splicing defects, and reduce the size and number of CGG-containing protein aggregates (Disney et al., 2012). Additional small molecules have also been identified to interact with CGG repeats, such as phospholipase A2 inhibitors (Qurashi et al., 2012), naphthyridine carbamate dimer (Hagihara et al., 2012), and piperine (Verma et al., 2019).

Compared with ASOs, small molecules bear an advantage over ASOs in that they are relatively smaller in size with a better blood-brain barrier permeability than ASOs. In addition, the small molecules can bind the CGG repeat RNAs without inhibiting translation of the downstream open reading frame. Studies have been conducted for two compounds to target CGG repeats in cellular models of FXTAS. The small molecules, 2′-O-methyl phosphorothioate, could modulate CGG toxicity better than oligonucleotides carrying 2′-O-methyl phosphorothioate (Tran et al., 2014). The identification of additional small molecules that target CGG repeats can help identify novel therapies for not only FXTAS, but for other related disorders mediated by CGG repeats like NIID.

Small molecules can be expanded as a nanoprobe to detect CGG repeats in the early diagnosis of CGG repeat disorders. For example, small molecules immobilized on the surface of carboxyl-functionalized Fe3O4 magnetic nanoparticles was reported for CGG trinucleotide repeat detection (Zhu et al., 2017). Overall, the design of CGG hairpin structure-specific small molecules may be of great therapeutic and diagnosis interest, but low binding specificity could lead to higher risks of off-target effects. Potential immune responses caused by small molecules could also be a concern.

### Gene Editing

First reported as a genome editor in eukaryotic cells in 2013 (Cong et al., 2013; Mali et al., 2013), Clustered Regularly Interspaced Short Palindromic Repeats (CRISPR), which form the basis of the bacterial immune system, has become a powerful gene editing strategy. Cas9 is an RNA-guided DNA nuclease that cleaves the DNA at a location specified by a guide RNA. CRISPR-based therapy is a promising strategy for many genetic disorders and already being assessed in clinical trials. A study from repeat expansion disorders suggests that the repeats can be excised from DNA by CRISPR/Cas9 technology (van Agtmaal et al., 2017).

Thus far, research on CRISPR-editing for CGG repeat diseases has focused on FXS, which could provide some insight for FXTAS and *NOTCH2NLC-*related disorders. Important progress has been made by using CRISPR/Cas9 to edit *FMR1* full mutation allele (CGG repeats >200) in FXS human iPSCs. It cleavages upstream of the CGG repeat site in *FMR1*, leading to the deletions of variable sizes, and finally resulting in the reactivation of *FMR1* expression and the elevation of FMRP levels (Park et al., 2015; Xie et al., 2016). In addition, the authors also reported a decline in DNA methylation at the *FMR1* locus in reactivated cells that produced FMRP. Inspired by this, another study used CRISPR/dCas9 to reverse the hypermethylation of CGG repeats in FXS iPSCs, and showed the reactivation of *FMR1* expression and phenotypic rescue (Liu et al., 2018). The same effect by the CRISPR/dCas9 system has also been verified in FXS embryonic stem cells (ESCs; Haenfler et al., 2018). Direct CGG repeat-targeting single-guide RNA (sgRNAs) appear to work better than promoter-targeting sgRNAs. Taken together, these data demonstrate that treatment of FXS could benefit from the excision of the expanded CGG repeats using CRISPR-editing.

Although this genome editing approach has great potential for the treatment of CGG repeat disorders, concerns remain. First, off-target effects are difficult to detect, leading to potential disturbance of the genome and the risk for permanent off-target genetic alterations at the DNA level. The presence of erroneous cleavage sites could be dependent on the repeat structure, gRNA, and target sequence. To overcome these shortcomings, a new modified RNA-targeting Cas9 (RCas9) system to target and track RNA was established (Nelles et al., 2016). The application of CRISPR/RCas9 resulted in efficient elimination of toxic CUG, CAG, CCUG, and GGGGCC repeat expansion RNAs in human cells along with the reversal of disease-related molecular defects like the reduction of polyglutamine proteins, likely making it more suitable for FXTAS. Further research is needed to verify the RNA-targeting CRISPR in the CGG repeat diseases.

The second issue is the virus-mediated delivery. Adeno Associated Virus (AAV) is the most common delivery vehicle *in vivo*. However, the carrying capacity of AAV is limited to drive gRNAs and Cas9 expression. Finally, the modified gRNA design should be optimized. Although the strategy is promising, many issues need to be resolved before proceeding to clinical applications.

### Perspectives

Recent years have seen a rise in findings on CGG/GGC repeat-related disorders. Various neurodegenerative diseases have been revealed to have the expanded CGG/GGC repeats within genes, like NIID and essential tremor. Those CGG/ GGC repeat disorders share lots of overlapping clinical and molecular phenotypes. For example, early FXTAS can resemble essential tremor to some extent. Thus, the abnormal repeat expansions may share similar underlying pathogenesis that affects multiple neurological phenotypes. Because underlying mechanisms and therapies for these *NOTCH2NLC-*related disorders are still poorly understood, correlates can be drawn from current research findings related to FXTAS. Moreover, these repeat-associated findings can be expanded to other microsatellite repeat expansion disorders beyond CGG/GGC repeat expansions. Several key questions in the field need to be addressed to provide clues for therapeutic approaches: (1) What are the exact mechanisms of the repeat RNAs and RAN translation that cause neuron toxicity? (2) Do the similar highly expressed CGG/GGC repeat transcripts in neurons play the same role in the pathogenesis contribution? and (3) Are there overlapping clinical features associated with the CGG/GGC expanded repeats in different diseases? Application of newly developed high-throughput technologies will facilitate the identification of the overlapping clinical features and mechanisms. This will undoubtedly provide better understanding of the repeat expansion diseases, offering new perspectives for therapeutic targets in this field.

## Author Contributions

KX and YL wrote the manuscript. EA and PJ edited the manuscript. All authors contributed to the article and approved the submitted version.

## Conflict of Interest

The authors declare that the research was conducted in the absence of any commercial or financial relationships that could be construed as a potential conflict of interest.
